# Trial Number and Sample Size Do Not Affect the Accuracy of the I2-Point Estimate for Testing Selection Bias Risk in Meta-Analyses

**DOI:** 10.7759/cureus.58961

**Published:** 2024-04-24

**Authors:** Steffen Mickenautsch, Veerasamy Yengopal

**Affiliations:** 1 Faculty of Dentistry, University of the Western Cape, Cape Town, ZAF; 2 Department of Community Dentistry, University of the Witwatersrand, Johannesburg, ZAF

**Keywords:** test accuracy, selection bias, meta-analysis, i2, bias testing

## Abstract

Aim

This study aims to establish the test sensitivity and specificity of the I^2^-point estimate for testing selection bias in meta-analyses under the condition of large versus small trial sample size and large versus small trial number in meta-analyses and to test the null hypotheses that the differences are not statistically significant.

Material and methods

Simulation trials were generated in MS Excel (Microsoft Corp., Redmond, WA), each consisting of a sequence of subject ID (accession) numbers representing trial subjects, a random sequence of allocation to group A or B, and a random sequence of a simulated baseline variable (“age”) per subject, ranging from 50 to 55. These simulation trials were included in five types of meta-analyses with large/small numbers of trials, as well as trials with large and small sample sizes. Half of the meta-analyses were artificially biased. All meta-analyses were tested using the I^2^-point estimate. The numbers of true positive (TP), false positive (FP), false negative (FN), and true negative (TN) test results were established. From these, the test sensitivity and specificity were computed for each of the meta-analysis types and compared.

Results

All non-biased meta-analyses yielded true negative, and all biased meta-analyses yielded true positive test results, regardless of trial number and trial sample size. No false positive or false negative test results were observed. Accordingly, test sensitivities and specificities of 100% for all meta-analysis types were established, and thus, both null hypotheses failed to be rejected.

Conclusion

The results suggest that trial number and sample size in a baseline variable meta-analysis do not affect the test accuracy of the I^2^-point estimate.

## Introduction

Meta-analyses form an important part of the methodology of systematic reviews, particularly that of randomized controlled trials (RCTs), with the purpose of statistically pooling the results of individual trials into one summary effect estimate. However, the internal validity of any meta-analysis result is directly dependent on that of its included trials. If the results of one or more single trial(s) are affected by selection bias, the result of the meta-analysis will be too.

Selection bias is present in an RCT when the random allocation of patients to the trial’s treatment groups is in some form subverted and patients with characteristics that are known to be conducive to a successful trial outcome are especially allocated to one treatment group only [1]. In theory, such subversion causes an imbalance in the measurements of baseline variables (such as the mean age of the patients per treatment group) in the trial. In contrast, in RCTs with true random allocation, such baseline measurements should differ between the treatment groups, due to the play of chance only. Consequently, a meta-analysis of such trials should show zero heterogeneity of the same baseline variables in-between trials and therefore reflect an I^2^ value of 0% [2]. The I^2^ statistic (measured in percentage) was developed for the purpose of estimating the proportion of variance in trial estimates that are due to heterogeneity. It ranges from 0% to 100% and is now commonly used as an integrated part of meta-analysis [3].

In 2014, Clark et al. reported that imbalances of baseline measurements in RCTs are reflected by an increased I^2^-point estimate when such measurements are statistically pooled with that of other trials [4]. On this basis, Hicks et al. (2018) presented a simple test that would assist in identifying selection bias in meta-analyses. The test includes the extraction of measurements (mean value, standard deviation {SD}, and sample size) for a baseline variable from all RCTs per treatment group that are included in a particular outcome meta-analysis and the calculation of the t-statistic for each trial. The baseline measurements are included in a fixed effect meta-analysis. If the result yields an I^2^-point estimate of >0%, the meta-analysis is repeated after the stepwise removal of trials with the largest t-statistic until reaching I^2^ = 0%. The outcome meta-analysis is then repeated without the removed RCTs and the new pooled effect estimate noted. If this effect estimate differs from the previous one, it is concluded that the outcome meta-analysis was affected by selection bias [2].

However, Rücker et al. (2008) established in a simulation study that the I^2^-point estimate increases with the number of subjects included in the trials in a meta-analysis. The authors observed that by artificially inflating the sample size under a random effects model meta-analysis, the I^2^-point estimate tended to be 100%, and thus, it is argued that I^2^ is of limited use in assessing heterogeneity [5].

In addition, von Hippel (2015) noted that the I^2^-point estimate can be biased when a meta-analysis includes too few trials. For example, the I^2^-point estimate of a small meta-analysis with seven trials for which true heterogeneity was absent overestimated the heterogeneity by an average of 12 percentage points and underestimated the heterogeneity by an average of 28 percentage points when true heterogeneity was actually present [3].

Against this background [3,5], it may be concluded that the I^2^-point estimate is not only imprecise due to its dependence on trial sample size but also easily affected by systematic error (bias) due to its dependence on the number of trials that are included in a meta-analysis. For that reason, it may be assumed that such a lack of precision and validity would negatively affect the accuracy of the I^2^-point estimate for selection bias testing of meta-analyses.

The aim of this simulation study was to establish the test’s sensitivity and specificity under the condition of large versus small trial sample size and large versus small trial number in meta-analysis and to test the null hypotheses that the differences were not statistically significant.

This manuscript has been made available online as preprint in Research Square (www.researchsquare.com: Mickenautsch S, Yengopal Y. Trial number and sample size do not affect accuracy of the I2- point estimate for testing selection bias risk in meta-analyses. A simulation study (preprint), 27 November 2023, preprint (version 1), available at Research Square {https://doi.org/10.21203/rs.3.rs-3658700/v1}).

## Materials and methods

The protocol of our study was made available online prior to its start [6].

Meta-analysis generation of simulation trials

Simulation trials were generated in MS Excel (Microsoft Corp., Redmond, WA), each consisting of three components, entered in the form of three parallel data columns: a sequence of subject ID (accession) numbers representing trial subjects, a random sequence of allocation to group A or B, and a sequence of a simulated baseline variable (“age”) per subject that was drawn randomly from a uniform distribution, ranging from 50 to 55, and stratified by ascending value.

The random allocation sequence was generated by block randomization with block size 4 using the “Sealed Envelope” online tool (Sealed Envelope Ltd., London, England) [7]. The sequence of a simulated baseline variable (“age”) per subject was generated using an online random number generator [8]. The comprehensive version of the generator was used for randomly selecting the values of the baseline variable for each subject with the following settings: lower limit = 50, upper limit = 55, allow duplication of results = yes, sort the results = ascend, and type of result to generate = integer.

These simulation trials were included in the following types of meta-analyses: type 1, large meta-analysis (N_Trials_ = 15) with large trials (N_Subjects _= 200 per trial); type 2, large meta-analysis (N_Trials_ = 15) with small trials (N_Subjects_ = 60 per trial); type 3, small meta-analysis (N_Trials_ = 5) with large trials (N_Subjects _= 200 per trial); and type 4, small meta-analysis (N_Trials_ = 5) with small trials (N_Subjects_ = 60 per trial).

The number of meta-analyses per type was determined by the use of sample size calculation according to the method by Buderer (1996) [9]. For each meta-analysis type, 50% of the meta-analyses were generated as affected by selection bias and 50% as non-biased. The former included two biased simulation trials. The trials were biased by sorting the subjects according to their baseline variable (“age”) in ascending order and assigning the first half with the lower values to group A and the other half with the higher values to group B.

In addition, meta-analysis type 4 was repeated with the difference that all biased trials were replaced with non-biased trials that included 200 subjects instead and thus formed meta-analysis type 5.

Sample size calculation

Sample size calculation was conducted using the online sample size calculator by Arifin (2023) [10] in line with the formula by Buderer (1996) [9]. The following settings were used: expected sensitivity and specificity for both = 80%, prevalence of disease (i.e., the prevalence of biased meta-analyses) = 50%, expected precision = 10%, confidence level 100 (1 - a) = 95%, and expected dropout rate = 0%. Accordingly, the calculation generated a required sample size of 123 meta-analyses for each meta-analysis type. In order to accommodate an even 50%/50% split between biased and non-biased meta-analyses, the number was increased to 124. Therefore, a total of 496 meta-analyses, with 124 for meta-analysis types 1-4, were generated. In addition, 62 meta-analyses were repeated, and 62 existing meta-analyses were altered by replacing biased small trials with non-biased large (N_Subjects _= 200) trials (meta-analysis type 5).

Meta-analysis of baseline variable and bias test

According to the test method presented by Hicks et al. (2018) [2], all meta-analyses of the baseline variable were conducted using the inverse variance method with a fixed effect model using the Review Manager (RevMan) 5.0.24 software (Cochrane, London, England). From each simulation trial, the following continuous data of the baseline variable (“age”) were calculated and entered per meta-analysis per groups A and B: mean value, standard deviation (SD), and sample size (N). After each meta-analysis was conducted, the resulting I^2^-point estimate was recorded.

Test accuracy measure

In order to investigate test accuracy, the numbers of true positive (TP), false positive (FP), false negative (FN), and true negative (TN) test results were established. These results were defined as follows: TP = biased meta-analysis with point estimate I^2^ > 0, FP = non-biased meta-analysis with point estimate I^2^ > 0, TN = non-biased meta-analysis with point estimate I^2^ = 0, and FN = biased meta-analysis with point estimate I^2^ = 0.

From the TP, FP, TN, and FN values, the sensitivity and specificity with a 95% confidence interval (CI) were computed for each meta-analysis type.

The test sensitivity was defined as the proportion of all meta-analyses with bias that yielded a positive test result (calculated as TP / (TP + FN)). The test specificity was defined as the proportion of all meta-analyses without bias that yielded a negative test result (calculated as TN / (FP + TN)) [11].

Statistical comparisons

It was planned to statistically compare the established test sensitivities and specificities between (i) large and small meta-analyses, meta-analysis type 4 versus type 2 and meta-analysis type 3 versus type 1; (ii) meta-analyses with large and small trials, meta-analysis type 4 versus type 3 and meta-analysis type 2 versus type 1; and (iii) non-biased small meta-analyses with small trials versus non-biased small meta-analyses with a mix of small and large trials, meta-analysis type 5 versus type 4, by means of the z-test for proportions. The null hypotheses were to be tested to determine whether the sensitivities and specificities from large versus small meta-analyses and meta-analyses with large versus small trials did not significantly differ (p > 0.05). Alpha was set at 5%.

Secondary analysis

In order to explore the effect of trial number and trial sample size in meta-analyses on I^2^-point estimate values in more detail, all generated point estimates were recorded and the mean values with standard deviation (SD) for meta-analysis types 1-4 calculated. From these values, the mean difference (MD) with 95% CI was computed for the following four comparisons: (i) meta-analyses with small versus large trial numbers, meta-analysis type 4 versus type 2 and meta-analysis type 3 versus type 1, and (ii) meta-analyses with small versus large trial sample size, meta-analysis type 4 versus type 3 and meta-analysis type 2 versus type 1.

Secondary analysis was not part of the study methodology per the original protocol [6] but was added after the results of the main analysis had become apparent.

## Results

Main analysis results

A total of 558 meta-analyses were generated, and 62 were repeated (Appendices {supplementary files/parts 1 and 2}). Of these, 248 contained two artificially biased trials. All non-biased meta-analyses yielded true negative (TN), and all biased meta-analyses yielded true positive (TP) test results, regardless of the trial number and trial sample size. Within the set parameters of this stimulation study, no false positive (FP) or false negative (FN) test results were observed (Appendices {supplementary files/part 3}). Accordingly, a 100% test sensitivity and specificity for meta-analysis types 1-4 were established. None of the non-biased meta-analysis type 5 yielded any positive test result; therefore, only the test specificity could be computed, which also yielded 100% (Table 1).

**Table 1:** Test sensitivity and specificity per meta-analysis type SS, test sensitivity; SP, test specificity; CI, confidence interval; N_Trials_, number of trials; N_Subjects_, sample size

Meta-analysis type	N_Trials_	N_Subjects_	SS (%)	95% CI	SP (%)	95% CI
1	15	200	100	94.2-100	100	94.2-100
2	15	60	100	94.2-100	100	94.2-100
3	5	200	100	94.2-100	100	94.2-100
4	5	60	100	94.2-100	100	94.2-100
5	5	60 and 200	Not estimable		100	97.1-100

Because all meta-analysis types indicated results of 100%, no statistical comparisons between test sensitivities and specificities were deemed necessary, and all null hypotheses, concerning test accuracy under the condition of large versus small trial number and large versus small trial sample sizes in meta-analysis, failed to be rejected.

Secondary analysis results

**Figure 1:**
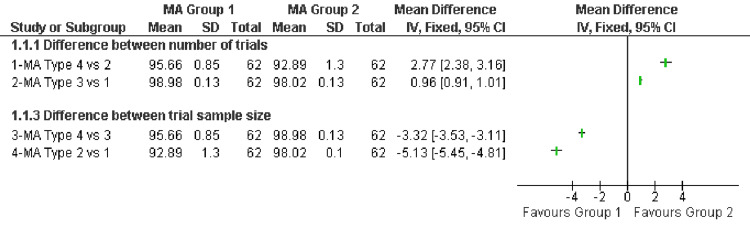
Comparison between mean I2-point estimates (SD) per type of meta-analysis MA, meta-analysis; SD, standard deviation; IV, inverse variance; CI, confidence interval

All non-biased meta-analyses of all meta-analysis types yielded a zero I^2^-point estimate. All biased meta-analyses yielded a point estimate of I^2^ > 0, which differed for each meta-analysis type (Appendices {supplementary files/part 2}). The mean I^2^-point estimates (SD) per type of meta-analysis are presented in Figure 1.

Biased meta-analyses with large trial numbers (15 trials/types 1 and 2) had statistically significantly higher I^2^-point estimates than meta-analyses with small trial numbers (five trials/types 3 and 4). This effect was larger when the sample size of the trials was small (60 subjects/type 4 versus type 2), MD: 2.77 and 95% CI: 2.38-3.16, than when the sample size was large (200 subjects/type 3 versus type 1), MD: 0.96 and 95% CI: 0.91-1.01.

Biased meta-analyses with small sample sizes (60 subjects/types 2 and 4) had statistically significantly lower I^2^-point estimates than meta-analyses with large sample sizes (200 subjects/types 1 and 3). This effect was larger when the trial numbers of the meta-analyses were large (15 trials/type 2 versus type 1), MD: -5.13 and 95% CI: -5.45 to -4.80, than when the trial numbers were small (five trials/type 4 versus type 3), MD: -3.32 and 95% CI: -3.53 to -3.11 (Figure 1).

## Discussion

The aim of this simulation study was to establish the sensitivity and specificity of the I^2^-point estimate for testing selection bias risk in meta-analyses under the condition of large versus small numbers of trials and large versus small sample sizes of trials included in such meta-analyses and to test the null hypotheses that the differences were not statistically significant.

The results of our study suggest that neither test sensitivity nor specificity of the I^2^-point estimate was affected by the trial number and sample size, and thus, all null hypotheses failed to be rejected.

Discussion of study results

It has been argued that the I^2^-point estimate increases as the median sample size increases [5], and therefore, removing trials until I^2^ is reduced to zero, according to the test by Hicks et al. (2018) [2], may not reflect the true reduction of selection bias in meta-analysis results. Accordingly, it may be assumed that any high-point estimates could be due to high sample sizes alone instead of selection bias and that the removal of a trial with a large sample size would be sufficient to reduce the I^2^-point estimate and thus artificially indicate non-biased status [12].

The results of our simulation study indicate that the baseline variable meta-analyses of trials that were included in an outcome meta-analysis will always reflect a zero I^2^-point estimate, as long as the allocation of these baseline variables in all trials followed a true random sequence, regardless of the sample size of the included trials. Therefore, all simulated meta-analyses with non-biased trials, including 200 subjects (types 1 and 3), generated the same zero I^2^-point estimate as simulated meta-analyses with trials that included 60 subjects (types 2 and 4) only. In addition, all meta-analyses that included a mix of non-biased trials with 60 or 200 subjects (type 5) also yielded zero-point estimates (Appendices {supplementary file/part 2}). Hence, the selection bias test indicated correct test specificities in all cases, regardless of the different trial sample sizes (Table 1).

In contrast, when the allocation was biased in some of the trials, the I^2^-point estimate reflected a value of above zero. Secondary analysis showed that these values varied, depending on the trial sample size: meta-analyses with small trials had lower I^2^-point estimates than meta-analyses with larger trials (Figure 1). This observation is in line with the results of Rücker et al.’s simulation study that the I^2^-point estimate increases with the number of trial subjects [5].

It has further been demonstrated that the I^2^-point estimate has a substantial positive bias when the number of trials included in a meta-analysis is small [3]. Nonetheless, the results of our study showed that the I^2^-point estimate of the baseline variable meta-analyses remains zero, provided that the allocation of these variables in all trials remains truly random. The simulated meta-analyses with non-biased trials, including 15 trials (types 1 and 2), generated the same zero values of the I^2^-point estimate as the simulated meta-analyses with five trials (types 3 and 4) only (Table 1).

In the cases where trials with biased allocation were included, the I^2^-point estimate reflected a >0 value. Secondary analysis showed that these values did vary relative to the trial number in line with the observation by von Hippel [3]: meta-analyses with 15 trials had larger I^2^-point estimates than meta-analyses with five trials (Figure 1).

The findings of our simulation study may be explained on the basis that the I^2^-point estimate of the baseline variable and not that of outcome meta-analyses was considered. A true random allocation of any baseline variable in all trials and with any influence due to the play of chance being excluded appears to assure a zero I^2^-point estimate, independent of the trial number and sample size. If such baseline variable from all trials was pooled in a fixed effect meta-analysis, the resulting I^2^-point estimate would reflect such underlying true random allocation, and thus, the selection bias test, with an I^2^ = 0 cutoff point, would yield a true negative (TN) result. However, as soon as the true random allocation of a baseline variable to trial treatment groups has been subverted, the I^2^-point estimate of a baseline variable meta-analysis will reflect a >0 value. Trial number and sample size will then affect such value, as reported elsewhere [3,5]. Nonetheless, with an I^2^ = 0 cutoff point, the bias test will yield a positive result, regardless of the value (by trial number and sample size affected) of a nonzero I^2^-point estimate.

Limitations of study design

Our study investigated only the effect of trial number and sample size on test accuracy. It particularly intended to exclude the effect of the play of chance on the I^2^-point estimate by stratifying the baseline variable (“age”) in ascending order prior to random allocation to treatment groups. In that way, we sufficiently excluded the play of chance as a possible confounding factor for the effect of trial number and sample size. Matching a random sequence of baseline variables with the random allocation (A and B) sequence would have included the play of chance and would have reduced the 100% test accuracy (sensitivity and specificity) to a more realistic level. However, it also would have prevented our study from establishing the actual effect of trial number and sample size on test accuracy.

For the same reason, we kept the simulated bias effect constant by including only two biased trials in all the different meta-analysis types. While this also assisted in ascertaining the actual effect of trial number and sample size, our study does not provide information concerning the effect of varying bias strength nor the influence of bias from more than one potential source.

How much the exclusion of the play of chance, the use of only two biased trials for biasing the meta-analyses, and the omission to consider any possible effect of varying/multiple biases would have affected I^2^-point estimate values is a matter of further research. Since our study established that sample size and trial number in baseline variable meta-analyses have no effect when the cutoff point is set at I^2^ = 0, a subsequent study could use our original simulation settings to generate control data against which results from new test settings that do simulate the play of chance, varying numbers of biased trials, and varying strength of multiple biases can be compared. In this way, their possible effects could be ascertained. It is recommended that these aspects be included in further simulation studies to the topic, as well as the comparison of the I^2^-point estimate to that of an absolute heterogeneity measure, such as tau-square (t^2^).

## Conclusions

The results of our study suggest that trial number and sample size in a baseline variable meta-analysis do not affect the test accuracy of the I^2^-point estimate with I^2^ = 0 as a cutoff point. These findings are important because they alleviate traditional reservations against the point estimate and thus allow its use for a more efficient and quantitative appraisal of selection bias risk in meta-analyses of clinical trials. Our results do not negate previously observed limitations of the I^2^-point estimate, which remain valid for measuring heterogeneity in outcome meta-analyses but appear not to be relevant in the baseline variable meta-analyses for selection bias appraisal with a zero I^2^-point estimate as cutoff.

Future simulation studies should investigate the combined effect of bias together with the play of chance on the overall test accuracy, as well as the comparison of the I^2^-point estimate to that of an absolute heterogeneity measure, such as tau-square (t^2^).

## Appendices

All data are fully available without restriction via https://data.mendeley.com/datasets/8y7ct6d5hk/1.
